# Group medical visits in the follow-up of women with a *BRCA *mutation: design of a randomized controlled trial

**DOI:** 10.1186/1472-6874-11-39

**Published:** 2011-08-24

**Authors:** Annemiek Visser, Judith B Prins, Nicoline Hoogerbrugge, Hanneke WM van Laarhoven

**Affiliations:** 1 Department of Medical Oncology, Radboud University Nijmegen Medical Centre, Nijmegen, The Netherlands; 2 Department of Medical Psychology, Radboud University Nijmegen Medical Centre, Nijmegen, The Netherlands; 3 Department of Human Genetics, Radboud University Nijmegen Medical Centre, Nijmegen, The Netherlands

## Abstract

**Background:**

*BRCA *mutation carriers have a 40-80% life-time risk of developing breast cancer. They may opt for yearly breast cancer surveillance or for prophylactic mastectomy. Both options show increased survival rates. It is a complex choice to be made between these two options. As a result most women experience high levels of distress and high needs for information. To fulfill the needs for psychosocial support and information we have introduced group medical consultations (GMCs). A GMC provides individual medical visits conducted within a group. This 90 minute group-visit with 8-12 patients gives patients the opportunity to spend more time with their clinician and a behavioral health professional and learn from other patients experiencing similar topics. However, it should be noted that group sessions may increase fear in some patients or influence their decision making.

**Methods/design:**

In this randomized controlled trial, 160 BRCA mutation carriers diagnosed maximally 2 years ago are recruited from the Radboud University Nijmegen Medical Centre. Participants are randomized in a 1:1 ratio to either the GMC intervention group (onetime participation in a GMC instead of a standard individual visit) or to a usual care control group. Primary outcome measures are empowerment and psychological distress (SCL 90). Secondary outcome measures are fear of cancer, information needs before the consultation and the received information, self-examination of the breasts, patient satisfaction, quality of life and cost-effectiveness. Data are collected via self-reported questionnaires 1 week before the visit, and at 1 week and at 3 months follow-up. A pilot study was conducted to test all procedures and questionnaires.

**Discussion:**

The possibility for interaction with other BRCA mutation carriers within a medical visit is unique. This study will assess the effectiveness of GMCs for BRCA mutation carriers to improve empowerment and decrease distress compared to individual visits. If GMCs prove to be effective and efficient, implementation of GMCs in regular care for BRCA mutation carriers will be recommended.

**Trial registration:**

The study is registered at ClinicalTrials.gov (NCT01329068)

## Background

*BRCA *mutation carriers have a high life-time risk of developing breast cancer and also of ovarian cancer. In a large meta-analysis of ten studies the mean cumulative breast cancer risk at 70 years was 46-80% for *BRCA1 *mutation carriers and 40-80% for *BRCA2 *mutation carriers [1-3]. Because of this high risk of breast cancer, women with a mutation in either the *BRCA1 *or *BRCA2 *gene are offered breast cancer surveillance, which includes annual clinical breast examination, annual mammography and annual contrast enhanced magnetic resonance imaging (MRI) of the breast [4-6]. Alternatively, women may opt for prophylactic bilateral mastectomy with or without breast reconstruction [7-10]. Prophylactic bilateral mastectomy reduces the risk for breast cancer by 89.5-100%[11], making yearly surveillance unnecessary. Although prophylactic mastectomy minimizes the risk of breast cancer, comparable survival rates are seen for prophylactic mastectomy and yearly breast cancer surveillance [10].

*BRCA *mutation carriers who underwent treatment for breast cancer before, have to deal with a 50-64% lifetime risk for developing a second primary breast cancer [12]. Most studies among *BRCA *mutation carriers who had breast cancer have shown a reduction in the risk of contralateral breast cancer, while results on survival rates were diverse [10,13-15]. Besides, the decision for contralateral prophylactic mastectomy depends on age, type of initial breast cancer surgery, prophylactic oophorectomy and the patient's opinion [16].

Thus, *BRCA *mutation carriers face a complex choice between these options. The knowledge of *BRCA *carriership and the consequently increased risk of developing breast (and ovarian) cancer may have a large psychosocial impact on mutation carriers. The level of cancer related distress among Jewish women increased significantly after the diagnosis of a *BRCA *mutation [17]. In another study 36 percent of women diagnosed with a *BRCA1 *mutation appeared to be sad or crying [18]. Several studies in the Netherlands showed that genetic testing, regardless of the test results may already increase levels of distress [19,20].

The provision of psychosocial support is intrinsically connected to the provision of guidance in the choice between surveillance and a prophylactic mastectomy. In a study of 233 women who were awaiting their initial appointments for risk assessment, consideration of prophylactic mastectomy strongly correlated with high levels of breast cancer anxiety and overestimation of one's breast cancer risk, whereas there was no association with objective breast cancer risk [21]. Recently, it was suggested that standard visit with a psychologist for high-risk women considering prophylactic mastectomy may be indicated since anxiety is one of the main reasons for considering a prophylactic mastectomy, and depression and grief were present in a third of the participating women [22]. Interestingly, in this study uncertainty about surgery and the need for further information were the reasons given most frequently for postponing prophylactic mastectomy, indicating that there is a great need for information in this group [23]. Generally, the decision about surveillance or prophylactic mastectomy is not a medically urgent one and women can indeed take time to gather and process all information and talk about it with others. Of note, patient satisfaction is an important predictor of quality of life, treatment compliance and lower rates of anxiety and depression [24]. In previous studies it has been shown that in order to improve the patient satisfaction it is important for the clinician to discuss psychosocial factors and to be responsive to the patients concerns [25,26].

To fulfill the needs just mentioned concerning psychosocial support and information we have introduced group medical consultations (GMCs) for the surveillance of *BRCA *mutation carriers. A GMC provides individual medical visits conducted within a group, giving patients the opportunity to spend more time with their own clinician in the setting of a support group with a behavioral health professional and learn from other patients experiencing similar topics. In contrast to the typical 15-20-minute office visit, a 90-minute group-visit with 8-12 patients permits ample time for discussion and education. The incorporation of the individual medical visit in a GMC distinguishes GMCs from educational workshops and support- and self-management groups. In general, the group of patients in a GMC is composed of different participants from meeting to meeting. Currently, GMCs have been offered to patients of different ages with a variety of diseases [27,28]. Although several benefits of GMCs compared to individual visits have been reported, the effects have not been evaluated for the surveillance of *BRCA *mutation carriers.

Most previous studies showed improved satisfaction in patients receiving GMCs and in health professionals providing GMCs. The unhurriedness of the clinician and the time spent with the clinician is appreciated most by patients [29-33]. Clinicians were positive about the innovative and multidisciplinary working method [34]. Besides improved quality of life, which is reported in several studies [32,35,36], also improved health behavior, self-efficacy for following certain health promoting interventions and knowledge of the disease was shown among diabetes patients and patients with coronary artery disease who participated in a group visit [35,37]. Also, quality of care is improved. For example, in a study of diabetic patients group visit participation was positively associated with patients receiving preventive procedures, review of medication and microalbuminuria test recorded in the registry [38]. Other studies in diabetic patients have confirmed these results [31,39-41]. When planned carefully, this type of visits may increase clinician satisfaction and productivity [30,36,42-44].

However, it should be noted that group sessions may not be beneficial [45]. Recently the effects of educational-support groups for *BRCA *mutation carriers have been studied [46]. This program included eight sessions with alternately a psychosocial (5 sessions) and a medical information focus (3 sessions); no individual visits were provided in the group. The study provided presumptive evidence that educational-support group participants were more inclined to undergo prophylactic mastectomy than non-participants. Possibly the occurrence of group members getting cancer during surveillance affected other group members. Group participation may have triggered feelings of anticipated regret, i.e. regret that women think they would have if they were to be diagnosed with breast cancer after not (yet) having chosen the option of prophylactic mastectomy [47]. Therefore, before introducing GMCs in regular patient care for *BRCA *mutation carriers the pros and cons of GMCs for this population should carefully be evaluated.

## Methods/Design

### Design

The study examines the effect of GMCs on patient empowerment and psychological distress of *BRCA *mutation carriers as well as several secondary outcome measures. This randomized controlled trial (RCT) will compare two groups; the intervention group, who will participate in a GMC once and the control group, who will receive care as usual (surveillance in an individual visit).

### Ethical consideration

The study has been approved by the medical ethical committee of the Radboud University Nijmegen Medical Centre. Full medical ethical approval has been obtained in February 2011.

### Study sample

A total of 160 recently diagnosed *BRCA *mutation carriers will be invited to participate in the study. To stimulate interaction and discussion within the group it is important to compose heterogeneous groups. Nevertheless, some homogeneity is needed to keep the GMCs relevant for everyone [34]. Therefore the following in- and exclusion criteria were formulated. Inclusion criteria are: carrier of a *BRCA1 *or a *BRCA2 *mutation; diagnosed maximally 2 years ago; and a minimum age of 25. Exclusion criteria are: currently involved in a diagnostic work-up because of a suspicion of breast cancer, either primary or metastatic; a history of prophylactic mastectomy or current psychiatric disease precluding visits in a group; insufficient command of the Dutch language to be able to follow a group discussion and fill out questionnaires in Dutch.

### Recruitment and randomization

**Figure 1:**
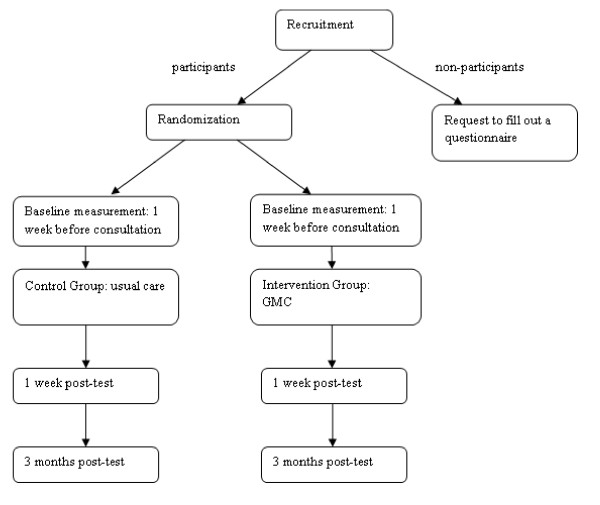
**Flow-chart of the study**.

Women with the *BRCA *mutation having their yearly surveillance in the Radboud University Nijmegen Medical Centre will be informed about the study during an individual visit to the outpatient clinic or approached via a telephone call by the researcher. As shown in Figure 1 patients will be randomized for their next visit to either the intervention group (GMC) or the control group (individual visit) after informed consent is obtained. Randomization will take place per patient in a 1:1 ration and in blocks of 16 patients. The individual clinician, will meet the same number of patients in a GMC and in an individual consultation, therefore we pre-stratified for clinician.

### Intervention

Patients randomized to the intervention group will participate once in a GMC instead of a standard individual visit. The GMC will provide individual medical visits conducted within a group, giving patients the opportunity to spend more time with their own clinician in the setting of a support group. In this way patients can learn from other patient's experiences with similar topics. A social worker will be present during every GMC. Besides leading the discussion and making sure every patient receives equal attention from the clinician, the social worker will be available for psychosocial related questions and support. The group visit itself will take 90 minutes. In advance, half an hour will be scheduled for physical examination, where privacy concerns will be taken into account. The control group will have their usual individual visit with the clinician. The clinician and the social worker will both use protocols with information based on most up-to-date publications concerning *BRCA *gene mutation related topics, specially developed for the GMCs.

### Study outcomes

At baseline, 1 week after and 3 months after the (group or individual) visit participants will be asked to fill out a questionnaire. Patients refusing participation will be asked to fill out one questionnaire to gain insight in reasons for not participating. Demographical data will be collected as well as the following measures.

### Primary outcome measures

Empowerment is the first primary outcome, which will be measured using the Empowerment Questionnaire for breast cancer patients. This questionnaire has been developed and validated in the Netherlands for the assessment of empowerment in psychiatric patients. With minor revisions, the 40-item questionnaire was validated in breast cancer survivors. The study involved 140 non-metastatic breast cancer patients who were treated in the past with curative intent. Four factors were extracted, explaining 53 percent of the total variance. Factor 1 was labeled 'Personal Strength', factor 2 'Social support', factor 3 'Community' and at least factor 4 'Health Care'. The internal consistency reliability for each factor demonstrated good reliability for all four factors and for the total empowerment scale (alpha = .94)[48].

Another primary outcome is psychological distress measured by the Symptom Checklist-90 (SCL-90). SCL-90 is designed to evaluate a broad range of psychological problems and symptoms of psychopathology. It can be used for measuring the progress and outcome of psychiatric and psychological treatments [45,49]. It consists of 90 items, yielding nine scores along primary symptom dimensions and three scores among global distress indices. A countless number of studies demonstrated the reliability, validity and utility of the instrument.

### Secondary outcome measures

Besides the primary outcomes, the following secondary outcomes are measured. The Cancer Worry Scale will be used to assess the fear of cancer. It consists of 8 items and is used in several studies among cancer patients. The internal consistency is good (alpha = .80)[50,51].

The information needs before the consultation and the actually received information after the consultation will be measured by a list of relevant topics for BRCA mutation carriers, including topics like prophylactic mastectomy, use of oral contraceptives, depressed mood. Participants can also add their own topics. In advance of the visit participant are asked to fill out which topics they want to discuss during the consultation. One week after the visit participants will indicate on the topic list which topics have actually been discussed.

Self-examination of the breasts will be measured by a question about the frequency of self-examination and about the reason not to examine their own breasts. One question is included to assess the patient's choice for a prophylactic mastectomy or surveillance.

To assess the patient satisfaction a combination of the QUOTE questionnaire [52] and some GMC specific questions will be used [34]. Overall satisfaction with the visit will be rated on a scale from 1 (not satisfied at all) to 5 (very satisfied).

The quality of life will be measured by the EORTC-Q30 and the EORTC-BR23. The EORTC-Q30 is a health-related quality of life questionnaire which is validated for oncological clinical research [53]. Chronbach's alpha was close to or higher than 0.70 for seven of the nine scales and ranged between 0.19-0.92 [54]. The questionnaire consists of 30 items, including a functional, symptom and health-related quality of life subscale. The EORTC-BR23 is a validated breast cancer-specific questionnaire [55,56]. The questionnaire consists of 23 items, including side effects of therapy, body image, sexuality and outlook for the future.

To evaluate the cost-effectiveness of the intervention costs will be measured by several questions from the Trimbos/iMTA questionnaire for Costs associated with Psychiatric Illness (TiC-P). This is a frequently used instrument for economic evaluations in mental health care [57].

The researcher will be present during the GMC for measuring the time spend per patient. This measure will be used in the analyses to test the relation between time spend per patient and the effectiveness of the GMC. Besides, topics discussed during the visits will be measured by the researcher.

### Power calculations

The primary aim of GMCs is to increase patient empowerment and to prevent an increase of psychological distress. Data on these outcome measures for *BRCA *mutation carriers are not yet available, therefore data from a rather comparable population, breast cancer patients, is used for the power analysis. Also, data of the SCL-90 is more widespread compared to empowerment data for breast cancer patients, therefore results of this questionnaire have been used. A healthy score on the SCL-90 ranges between 90-130, while breast cancer patients, one year or longer after curative treatment for breast cancer, achieved an average score of 138.5 [58]. In this study we strive for a high normal average score of 125. This implies that an average difference of 13.5 points can be defined as clinically relevant. The standard deviation is 45, which is based on three norm groups [49]. To assess a clinically relevant effect of GMC on psychological distress with a power of 80%, a two-side significance of 5%, a standard deviation of 45 and an expected drop-out of 15%, 80 patients need to be included in each group.

### Statistical analysis

For the primary analysis a covariance analysis (ANCOVA) will be conducted. Differences in empowerment and SCL-90 scores (primary outcome measures) at baseline and one week after the GMC between the intervention group and control group will be compared. In addition ANCOVA provides the opportunity to compare trends over time between the two groups. The SCL-90 scores at T1 or T2 will be included as dependent variables and the group (individual consult or GMC) as independent variable. Furthermore, the SCL-90 score at baseline will be a covariate. The analysis of the secondary outcome measures is a repeated measurement analysis (ANOVA or mixed model) to compare trends over time between the two groups.

### Cost-effectiveness analyses

Beside effectiveness of the intervention, another important measure is the cost-effectiveness. This study will compare the difference in total costs for health care and production losses between the intervention group and the control group. These costs will be estimated by several questions. The total direct medical costs (for example outpatient visits, length of stay in hospital, use of medication) will be calculated by the use of reference unit prices of health care services. The total estimated costs are rated against change in the level of distress.

### Preliminary results

Seven *BRCA *mutation carriers participated in a GMC in our pilot study. The overall patient satisfaction with the GMC was quite promising with an average score of 3.7 (sd = 1.1) on a range of 1-5. More than half of the participants (57.1%) would choose a GMC in the future again. 42.9% experienced support from other patients. A comparison of the topics, which are discussed during the GMC according to patients and according to health professionals showed that according to patients fewer topics were discussed in the GMC compared to the number of topics that health care workers reported (table 1). For instance, all care providers indicated that the risk for breast cancer and the results of the MRI and mammography were discussed, while less than half of the patients mentioned these topics.

**Table 1:** Topics discussed during GMC according to patients and to health care professionals

Topics	Care professionals (%) (n = 2)	Patients (%) (n = 7)
The risk of breast cancer	100	42.9
Self-examination of the breasts	100	100
Results from MRI and mammography	100	28.6
Control frequency in the hospital	100	71.4
Prophylactic mastectomy	100	57.1
Factors influencing the choice between prophylactic mastectomy or surveillance	100	57.1
Breast reconstruction	0	28.6
Breast enlargement	0	0
Controls of ovaries	0	0
Surgical removal of the ovaries	100	57.1
Use of birth control pills	0	0
Pregnancy	100	71.4
Breast feeding	100	71.4
Osteoporosis	0	14.3
Hot flashes	50	28.6
Situation of family members	100	100
Reaction of partner/family/friends on the diagnosis	0	28.6
Sad feelings	100	71.4
Feelings of fear	100	85.7
Contact with social work	0	14.3
Other topics	50	28.6

## Discussion

Several studies showed the advantages of GMCs compared to individual visits. However, for *BRCA *mutation carriers the effectiveness has never been studied. Since GMCs may have disadvantages the effects of GMCs should carefully be studied.

Most research among other patient groups showed positive effects on patient and caregiver's satisfaction. The participants in the pilot study were also satisfied about the GMC. This is of importance as a high patient satisfaction is related to a high quality of life, more compliance and less anxiety and depression [24].

We observed a remarkable difference in the number of topics discussed during the GMC according to patients and according to health care workers. This may be explained by the fact that patients remember only 20-60% of the information in medical visits and only remember information that is relevant to them at that moment. The working memory is limited, therefore only relevant information will be processed and stored [59]. It should be noted that *BRCA *mutation carriers have a large need for information [22].

GMCs give the opportunity to provide this information in a relatively short amount of time, while patients can make a selection of information, which is particularly relevant for their own situation. Besides, we expect information gathered from fellow patients to be better remembered. At the same time any incorrect information given by other patients can be corrected immediately by the health care professional to prevent misunderstandings.

GMCs may fulfill the need for extra attention for psychosocial related problems, both by health care providers and by fellow patients. The possibility for interaction with other *BRCA *mutation carriers within a medical visit is unique.

Of note, despite the widespread implementation of GMCs in different patient groups, the cost-effectiveness remains unproven. In the current health care system cost effectiveness is an important factor to take into account too.

In conclusion, the aim of our study is to assess the effectiveness of GMCs for *BRCA *mutation carriers to improve empowerment and decrease distress compared to individual visits. If GMCs prove to be effective and efficient, implementation of GMCs in regular care for *BRCA *mutation carriers will be recommended.

## Competing interests

The authors declare that they have no competing interests.

## Authors' contributions

JBP and NH have contributed to the study protocol and revised the manuscript. HWML has also contributed to the study protocol, conducted the pilot study, performed analysis of the pilot data, and revised the manuscript. AV has contributed to the study protocol, performed analysis of the pilot data and wrote the manuscript. All authors read and approved the final manuscript.

## Pre-publication history

The pre-publication history for this paper can be accessed here:

http://www.biomedcentral.com/1472-6874/11/39/prepub
